# A Novel Homozygous Loss-of-Function Variant in *SPRED2* Causes Autosomal Recessive Noonan-like Syndrome

**DOI:** 10.3390/genes15010032

**Published:** 2023-12-25

**Authors:** Maria Elena Onore, Martina Caiazza, Antonella Farina, Gioacchino Scarano, Alberto Budillon, Rossella Nicoletta Borrelli, Giuseppe Limongelli, Vincenzo Nigro, Giulio Piluso

**Affiliations:** 1 Department of Precision Medicine, University of Campania “Luigi Vanvitelli”, 80138 Naples, Italy; maria.elena.onore@gmail.com (M.E.O.); farina.a.93@gmail.com (A.F.); alberto.budillon@hotmail.it (A.B.); ronib@hotmail.it (R.N.B.); vincenzo.nigro@unicampania.it (V.N.); 2 Inherited and Rare Cardiovascular Diseases Unit, Department of Translational Medical Sciences, University of Campania “Luigi Vanvitelli”, Monaldi Hospital, 80131 Naples, Italy; martina.caiazza@yahoo.it (M.C.); gioac.scarano51@gmail.com (G.S.); giuseppe.limongelli@unicampania.it (G.L.); 3 Medical Genetics Unit, AORN “San Pio”, Hospital “G. Rummo”, 82100 Benevento, Italy; 4 Institute of Cardiovascular Science, University College London and St. Bartholomew’s Hospital, London E1 4NS, UK; 5 Telethon Institute of Genetics and Medicine (TIGEM), 80078 Pozzuoli, Italy

**Keywords:** Noonan syndrome, *SPRED2*, autosomal recessive inheritance, RASopathies

## Abstract

Noonan syndrome is an autosomal dominant developmental disorder characterized by peculiar facial dysmorphisms, short stature, congenital heart defects, and hypertrophic cardiomyopathy. In 2001, *PTPN11* was identified as the first Noonan syndrome gene and is responsible for the majority of Noonan syndrome cases. Over the years, several other genes involved in Noonan syndrome (*KRAS*, *SOS1*, *RAF1*, *MAP2K1*, *BRAF*, *NRAS*, *RIT1*, and *LZTR1*) have been identified, acting at different levels of the RAS-mitogen-activated protein kinase pathway. Recently, *SPRED2* was recognized as a novel Noonan syndrome gene with autosomal recessive inheritance, and only four families have been described to date. Here, we report the first Italian case, a one-year-old child with left ventricular hypertrophy, moderate pulmonary valve stenosis, and atrial septal defect, with a clinical suspicion of RASopathy supported by the presence of typical Noonan-like facial features and short stature. Exome sequencing identified a novel homozygous loss-of-function variant in the exon 3 of *SPRED2* (NM_181784.3:c.325del; p.Arg109Glufs*7), likely causing nonsense-mediated decay. Our results and the presented clinical data may help us to further understand and dissect the genetic heterogeneity of Noonan syndrome.

## 1. Introduction

RASopathies are a group of genetic syndromes caused by germline mutations in components or regulators of the RAS-mitogen-activated protein kinase (RAS-MAPK) pathway [1]. The main biological processes regulated by this signal transduction cascade are cell cycle regulation, differentiation, proliferation, apoptosis, and senescence [2]. Neurofibromatosis type 1 (NF1, MIM:162200), Noonan syndrome (NS, MIM: 163950), Noonan syndrome with multiple lentigines (formerly known as LEOPARD syndrome; NSML, MIM: 151100), Costello syndromes (CS, MIM:218040), Legius syndrome (LGSS, MIM:611431), cardiofaciocutaneous syndrome (CFC, MIM:115150), capillary malformation-arteriovenous malformation syndrome (CM-AVM, MIM: 608354), and Mazzanti syndrome (OMIM 607721 and 617506) are included in the group of RASopathies and are widely described as syndromes presenting a broad range of clinical manifestations with partially overlapping features.

Since 2001, mutations in more than 20 genes have been reported to cause RASopathies [1]. Pathogenic variants are reported in several genes encoding members of the RAS-MAPK pathway, including phosphatases (*PTPN11*), core components of the MAPK cascade (*BRAF*, *RAF1*, *MAP2K1*, *MAP2K2*, and *MAPK1*), members of the RAS subfamily of GTPases (*HRAS*, *NRAS*, *MRAS*, *RRAS*, *RRAS2*, and *RIT1*), and both negative (*NF1*, *LZTR1*, *CBL*, and *SPRED1*) and positive (*SOS1*, *SOS2*, *SHOC2*, and *PPP1CB*) regulators of Ras function [3]. Alterations in RAS-MAPK pathway signaling represent the underlying pathogenetic mechanism of RASopathies, mainly transmitted as dominant traits.

NS is a common developmental disorder with an autosomal dominant inheritance and is characterized by a peculiar face, short stature, and congenital heart defects, including pulmonary valve stenosis (PVS), cardiomyopathy, hypertrophic cardiomyopathy (HCM), and atrial septal defect (ASD) [4]. Other findings include cryptorchidism in males and an unusual chest shape with pectus carinatum or pectus excavatum [5].

In 2001, pathogenic variants in *PTPN11*, encoding the non-receptor protein tyrosine phosphatase SHP-2, were first associated with NS, and *PTPN11* is today the most commonly mutated NS gene [6]. Over the years, several other genes involved in NS have been identified, such as *KRAS*, *SOS1*, *RAF1*, *MAP2K1*, *BRAF*, *NRAS*, *RIT1*, and *LZTR1*. About 93% of heterozygous mutations causing NS involve *PTPN11*, *SOS1*, *RAF1*, and *RIT1* [4].

Very recently, loss-of-function (LoF) variants in *SPRED2* were recognized as causing NS with an autosomal recessive inheritance pattern (NS14; MIM: 619745) [7,8]. *SPRED2* is one of the three members of the Sprouty-related EVH-1 domain-containing (SPRED) family that negatively regulate the RAS-MAPK pathway [9]. The three family members—*SPRED1* (NM_152594.2), *SPRED2* (NM_181784.3), and *SPRED3* (NM_001394336)—are characterized by three shared domains: the N-terminal Ena/VASP homology 1 (EVH1) domain, the central c-KIT binding domain (KBD), and the C-terminal sprout (SPR), a cysteine-rich region. Two uncharacterized regions link the EVH1, KBD, and SPR domains [10,11].

Lacking enzymatic activity, SPRED proteins use their EVH1 domain to interact with neurofibromin, a RAS-GTPase activating protein crucial to inactivating the RAS-MAPK cascade, while the SPR domain is involved in membrane localization and is specifically required to recruit neurofibromin into the plasma membrane compartment to downregulate RAS signaling [12]. While the EVH1 and SPR domains are involved in inhibition of the MAPK cascade, the KBD domain of SPRED1 and SPRED2 is required to bind or become phosphorylated by c-KIT; however, the KBD domain results in inactivity in SPRED3 [10,13,14]. The SPRED proteins are encoded by three different genes (*SPRED1*, *SPRED2*, and *SPRED3*) located on chromosomes 15, 2, and 19 [14].

Heterozygous variants in *SPRED1* are associated with LGSS, an autosomal dominant disorder characterized by multiple café-au-lait spots, axillary freckling, and variable dysmorphic features such as hypertelorism or macrocephaly, mild learning disabilities, or attention problems, without cardiac involvement [15,16].

While LGSS is considered an NF1-like condition, without the neurofibromas or other tumor manifestations typical of NF1, the phenotype associated with LoF variants in *SPRED2* presents a clinical spectrum comparable to NS, suggesting that *SPRED1* and *SPRED2* play different roles in development [7]. To date, *SPRED3* has not been associated with RASopathies or other genetic syndromes.

Here we report the case of a one-year-old child with ventricular hypertrophy and clinical suspicion of RASopathy, in which whole exome sequencing (WES) identified a novel homozygous LoF variant in *SPRED2* (NM_181784.3:c.325del; p. Arg109Glufs*7). Our results and clinical data can help us further understand and distinguish between the genetic heterogeneity of NS.

## 2. Materials and Methods

### 2.1. Clinical Evaluation

The patient was evaluated at the Inherited and Rare Disease Unit, Monaldi Hospital, University of Campania “Luigi Vanvitelli”. A comprehensive clinical genetic evaluation and cardiological assessment was performed. The diagnosis of HCM was based on recent cardiomyopathy guidelines [17], which define HCM as unexplained left ventricular hypertrophy in the absence of other cardiac or systemic disease and left ventricular outflow tract obstruction (gradient at rest ≥30 mmHg or ≥50 mmHg with Valsalva).

### 2.2. Sample Collection

Written informed consent for blood sample collection and genetic investigation was obtained from the proband’s parents, according to the Declaration of Helsinki. For each subject, genomic DNA was extracted using standard procedures.

### 2.3. Whole Exome Sequencing

For the proband and his parents (family trio), exome sequencing was carried out using the Agilent SureSelectXT Human All Exon V8 kit (Agilent Technologies, Santa Clara, CA, USA), according to the manufacturer’s instructions. Sequencing was performed using the Novaseq 6000 system (Illumina, San Diego, CA, USA). The mean coverage of targeted regions was 99.3% at 10x, ensuring the detection of genetic variants with high sensitivity and specificity. Sequence reads were mapped to the reference human genome assembly (Dec. 2013, GRCh38/hg38) and analyzed using an in-house pipeline. The calling of single nucleotide variants (SNVs) and small insertions/deletions (Ins/Del) was performed with the Genome Analysis Toolkit (GATK; gatk.broadinstitute.org). Called SNVs and Ins/Del variants were annotated using the Ensembl Variant Effect Predictor [18]. For data filtering, we successively considered: (1) variants that passed quality control and with more than 10 reads; (2) variants with allele frequency <1% in global and European populations as reported in the Genome Aggregation Database (gnomad.broadinstitute.org); (3) variants that were not reported in our internal database of about 5000 exomes; (4) variants occurring *de novo* and all possible patterns of Mendelian inheritance; (5) variants occurring in genes already associated with RASopathies (virtual panels); (6) variants with a potential effect on gene function and predicted to be pathogenic/likely pathogenic (SIFT, PolyPhen, MutationTaster, PROVEAN, ClinVar). Candidate variants were classified in accordance with American College of Medical Genetics and Genomics (ACMG) guidelines [19].

### 2.4. Homozygosity Mapping

Homozygosity mapping was performed using AutoMap directly on VCF (Variant Call Format) calls from the proband’s WES data [20].

### 2.5. Variant Validation and Segregation Analysis

The causative variant in *SPRED2* was annotated according to the Human Genome Variation Society (HGVS) nomenclature on RefSeq NM_181784.3 [21]. After PCR amplification of exon 3 and its flanking regions (SPRED2_ex3_Forward: 5′-AGGGGTTAGAGGGGTTTTGG-3′; SPRED2_ex3_Reverse: 5′-GCATCTACTGACCTGGTCCC-3′), the variant was validated by segregation analysis in the proband and his parents. PCR products were double-strand sequenced using BigDye Terminator sequencing chemistry (Life Technologies, Carlsbad, CA, USA) and analyzed on an ABI 3130xL automatic DNA sequencer (Life Technologies).

## 3. Results

### 3.1. Case Presentation

The proband is a child of one year of age born to apparently unrelated, healthy parents. Pregnancy was uncomplicated, and morphologic echography at 20 weeks showed only mild right pyelectasis (5.8 mm). He was delivered at 29 weeks’ gestation by cesarean section after placental abruption. His birth weight was 1725 g (98th percentile; +2.01 SD); length and occipito-frontal circumference (OFC) were not reported. The Apgar score was 6 at 1 min and 7 at 5 min. At birth, he presented left ventricular hypertrophy and moderate PVS and ASD, and was therefore treated with propanolol (0.25 mg/kg/dose every 8 h) to improve heart function. Both his parents underwent a cardiological ultrasound examination, which did not show any sign of altered cardiac morphology or function. Physical examination at the age of 3 months (corrected gestational age of 41 weeks) revealed a hypomimic face, dysmorphic facial features, and bilateral convergent strabismus. Weight, length, and OFC were 3480 g (26th percentile; −0.67 SD), 52.2 cm (53rd percentile; +0.06 SD), and 36.5 cm (57th percentile; +0.68 SD), respectively.

**Figure 1:**
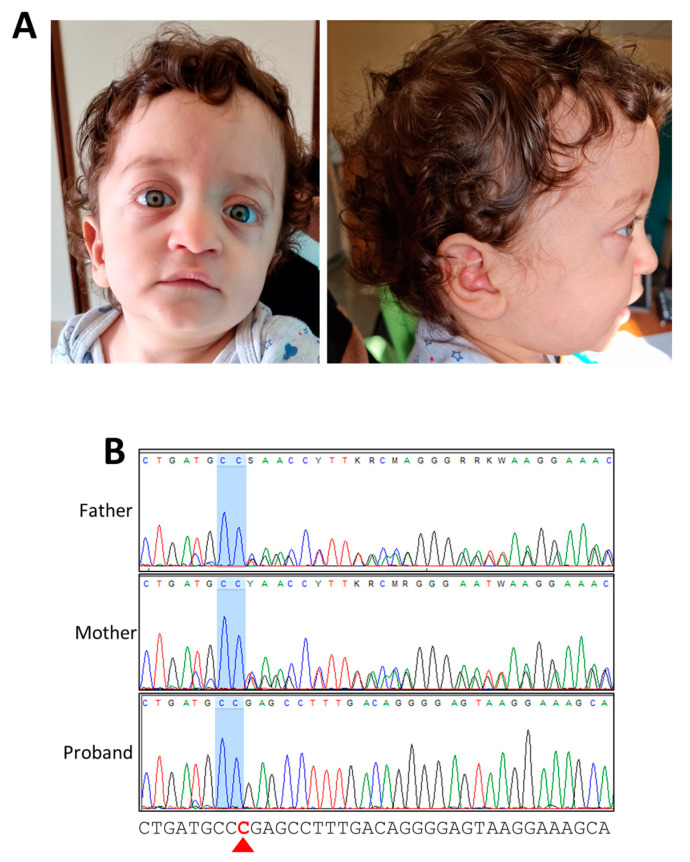
Clinical features of the proband and genetic results of the subjects examined. (**A**) Typical Noonan syndrome features observed in the proband: high forehead, bitemporal narrowing, low-set and posteriorly rotated ears, thick helix, hypertelorism, down-slanted palpebral fissures, prominent eyes, prominent nasal bridge, deep philtrum, large mouth, thin lips, pointed chin, mild micrognathia, and low posterior hairline. (**B**) Electropherograms confirming the homozygous NM_181784.3:c.325del variant in the proband and heterozygosity for the same variant in his father and mother. The red arrowhead indicates the deleted nucleotide at position 325.

At the last examination at 13 months, the weight was 7850 g (2nd percentile; −2.08 SD), the length was 71 cm (1st percentile; −2.44 SD), and the OFC was 47 cm (70th percentile; +0.51 SD). Clinical features included a high forehead, bitemporal narrowing, low-set and posteriorly rotated ears, a thick helix, hypertelorism, down-slanted palpebral fissures, prominent eyes, a prominent nasal bridge, a deep philtrum, a large mouth, thin lips, a pointed chin, mild micrognathia, a low posterior hairline, and a short and webbed neck (Figure 1A). As the child was still very young, we were unable to adequately evaluate his neurocognitive and language development.

### 3.2. Molecular Diagnosis

**Figure 2:**
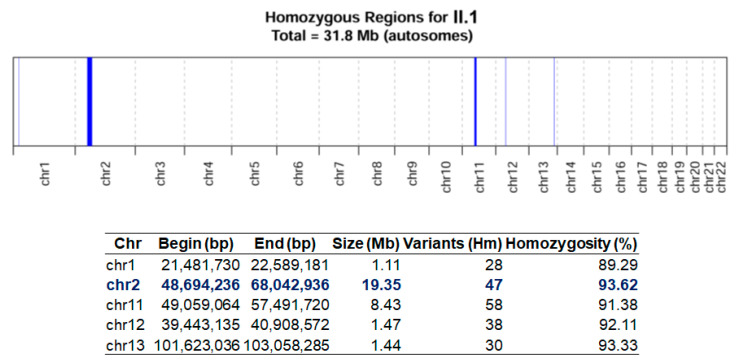
Homozygosity map of the proband. The bar graph (**top**) highlights in scale the homozygous regions detected for the autosomes, while the table (**bottom**) shows chromosomal position, size in Mb, and percentage of homozygosity for each interval. The region on chromosome 2, including *SPRED2,* is highlighted in blue.

WES analysis identified a novel homozygous 1 bp deletion in exon 3 of *SPRED2* (NM_181784.3:c.325del; p.Arg109Glufs*7), mapped on chromosome 2p14. This variant falls in the N-terminal EVH1 domain of *SPRED2* and is predicted to be likely pathogenic according to ACMG guidelines (PVS1, PM2), possibly causing nonsense-mediated decay. Further, this variant was not previously reported in any public databases such as LOVD (https://www.lovd.nl/; accessed on 1 November 2023) and ClinVar (https://www.ncbi.nlm.nih.gov/clinvar/; accessed on 1 November 2023) and was never found in GnomAD (https://gnomad.broadinstitute.org/; accessed on 1 November 2023). Segregation analysis confirmed homozygosity in the proband, while both healthy parents were heterozygous for this variant (Figure 1B). Although the parents came from a small town in Campania (Italy), they denied consanguinity. However, homozygosity mapping revealed homozygous regions for approximately 32 Mb on the proband’s autosomal chromosomes (Figure 2), corresponding to a homozygosity of about 1.06% of the whole genome [22,23]. The largest of these regions was 19.35 Mb on chromosome 2 (from position 48,694,236 to 68,042,936) and included *SPRED2*, suggesting possible common ancestors with a less than fifth degree relationship.

## 4. Discussion

NS, along with NF1, is one of the most common RASopathies, a group of developmental syndromes caused by mutations in genes encoding proteins involved in regulating the RAS-MAPK pathway. Noonan clinical features typically include facial dysmorphisms, such as triangular face, micrognathia, low-set/posteriorly rotated ears, and short and/or webbed neck; congenital cardiac defects (frequently PVS and ASD) and HCM are also present, as well as postnatal growth retardation, developmental and cognitive delay, and congenital hypotonia [24]. Following the initial description of *PTPN11* as an NS-associated gene [6], several other genes have been found mutated in NS patients, mainly with autosomal dominant inheritance [1] except for *LZTR1*, in which NS-associated variants with dominant (NS10; MIM 616564) and recessive (NS2; MIM 605275) inheritance patterns are reported [25,26].

More recently, bi-allelic LoF variants in *SPRED2* were recognized as causing an NS-like phenotype (NS14; MIM 619745), thus identifying the second recessively inherited NS gene [7]. To date, only four families and six affected individuals have been reported worldwide [7,8]. All subjects described were born to healthy consanguineous parents of Syrian, Tunisian, and Turkish origin. Here we report the first case of an Italian family with a homozygous LoF variant in *SPRED2*, with homozygosity mapping suggesting a possible common ancestor in the proband’s parents.

**Figure 3:**
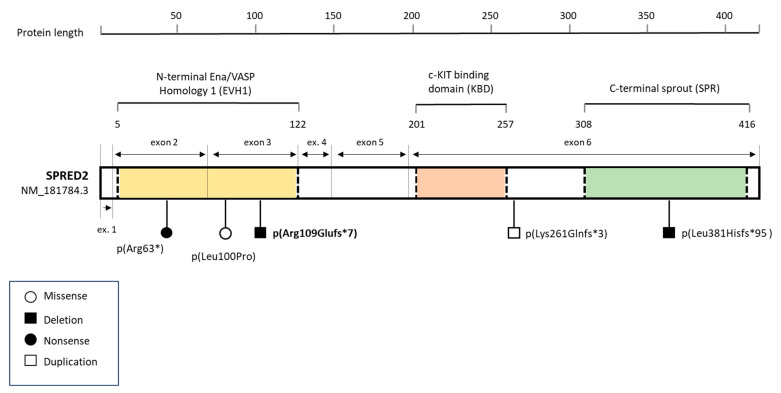
Graphical view of the SPRED2 protein showing its functional domains and published pathogenic variants reported to date. Functional motifs of SPRED2 are color-coded. Reported pathogenic variants are grouped by shape according to their functional effect. The variant identified in this study is highlighted in bold.

In the previously reported cases of NS14, four LoF variants in *SPRED2* were identified that fall within the different domains of the protein [EVH1 (n = 2), SPR (n = 1), and the uncharacterized region near c-KIT (n = 1)] (Figure 3). The variant identified in our index case (NM_181784.3:c.325del; p. Arg109Glufs*7) is also located in the N-terminal EVH1 domain (Figure 3).

Among the reported homozygous *SPRED2* variants, including the novel variant we identified, four out of five are predicted to be LoF with a deleterious effect on SPRED2 function and stability, whereas the p.Leu100Pro substitution is likely to result in a SPRED2 protein with reduced and/or less stable binding to neurofibromin, similarly reducing its function and stability [7].

Our patient and the six previously reported cases all present Noonan-like facial features, and the typical clinical manifestations occurred in the first decade of life (Figure 1A and Table 1). Growth retardation was observed in all subjects, while five out of seven had varying degrees of developmental delay, intellectual disability, and language impairment, which could not yet be assessed in our patient. Congenital heart defects, mainly PVS (5/7) and HCM (4/7), were also reported in all patients. Chest abnormalities are also typical in NS. Pectus carinatum was present in our patient, and pectus excavatum was present in five of the previously reported cases. Hyperlaxity was present in our patient and was also observed in four of the previously reported cases. Regarding the skin/ectodermal abnormalities, our patient presented with deep palmar creases, which have already been highlighted by Motta et al. in two of the reported cases of *SPRED2*-related NS. [7]. In addition, and similarly to the others, he did not show café-au-lait spots and freckling, which are not distinguishing clinical features, unlike in *SPRED1*-related LGGS.

In previously reported cases of *SPRED2*-related NS, as well as in the first Italian family described here, homozygosity for LoF variants is always present, and consanguinity is documented or strongly suspected. Thus, NS14 is most likely an extremely rare form of NS. Based on the limited number of patients with bi-allelic LoF variants in *SPRED2* reported to date, the pathogenic mechanism underlying the observed NS-like clinical presentation needs to be further investigated and clarified. *SPRED1* and *SPRED2* are both associated with monogenic conditions with different models of inheritance. The two genes seem to have similar expression patterns, are prevalent in embryonic (*SPRED1*) and adult (*SPRED2*) tissues, and dynamically regulate the RAS-MAPK pathway at different times and stages [7,27]. The haploinsufficiency characterizing *SPRED1*-related LGGS compared to LoF observed in *SPRED2*-related NS may suggest that SPRED1 likely has a primary role in the proper functional localization of neurofibromin-regulating MAPK signaling. Conversely, the similar function of SPRED2 could also be partially replaced by SPRED1, making SPRED2 suitable for a loss-of-function mechanism. However, further investigations will be necessary to better understand how mutations in these functionally similar genes contribute to such dissimilar phenotypes.

## Figures and Tables

**Table 1:** Bi-allelic *SPRED2* mutations and clinical phenotypes.

	Patient 1	Patient 2	Patient 3	Patient 4	Patient 5	Patient 6	Present Case
Reference	Motta et al. [7]				Markholt et al. [8]		This report
Ethnicity	Tunisian	Turkish	Turkish		Syrian		Italian
Parents	consanguineous	consanguineous	consanguineous		consanguineous		possible common ancestor
Sex, age at onset	F, 2 years	M, 19 mo	F, 2 years	M, n.r.	F, 3 years	F, 2 years	M, 2 mo
Age at last examination	11 years 11 mo	8 years	14 years 2 mo	39 years	4 years 1 mo	3 years 2 mo	1 year 2 mo
Height (SD)	136.5 cm (−1.50)	122.2 cm (−1.00)	145 cm (−2.41)	144 cm (−4.35)	89 cm (−3.2)	92.4 cm (−1.2)	71 cm (−2.44)
Weight (SD)	27.5 kg (−2.30)	22 kg (−1.09)	42 kg (−1.02)	56 kg (−1.29)	15 kg (−0.8)	13.6 kg (−0.5)	7.85 Kg (−2.08)
Head circumference (SD)	55 cm (+1.00)	51 cm (+0.86)	57 cm (+1.32)	56 cm (−1.00)	49 cm (−0.3)	51 cm (+0.8)	47 cm (+0.51)
**SPRED2 Variants**							
Domain	EVH1	EVH1	SRP		uncharacterized regions		EVH1
Zygosity	Hom	Hom	Hom		Hom		Hom
cDNA change (NM_181784.3)	c.187C>T	c.299T>C	c.1142_1143delTT		c.780dup		c.325del
AA change	p.Arg63*	p.Leu100Pro	p.Leu381Hisfs*95		p.Lys261Glnfs*3		p.Arg109Glufs*7
**Development**							
Developmental delay	no	mild	yes	yes	yes	n.r.	mild
Intellectual disability	yes	mild	mild	mild	yes	n.r.	too early to evaluate
Language delay	no	yes	yes	yes	yes	no	mild
Learning disorder	attention deficit	yes	yes	yes	yes	n.r.	too early to evaluate
**Neurological features**							
Hypotonia	no	during infancy	yes	yes	no	no	yes
**Cardiovascular features**							
Congenital heart defects	PVS	PVS, pulmonary balloon valvuloplasty, small secundum ASD	mild aortic insufficiency, mitral valve prolapse	no	yes	yes	mild pulmonary stenosis
Hypertrophic cardiomyopathy	no	asymmetrical hypertrophy of the interventricular septum	focal interventricular septum hypertrophy	yes	no	no	yes
**Skeletal features**							
Chest	pectus excavatum	superior pectus carinatum, inferior pectus excavatum	pectus excavatum	pectus excavatum	pectus excavatum	no	pectus carinatum
Hyperlaxity	yes	yes	yes	no	yes	no	yes
Other skeletal anomalies	kyphosis, clinodactyly, abnormal toe position	limited extension of elbows, cubitus valgus, winged shoulder blades, kyphosis, mild pes valgus and pes planus	cubitus valgus	no	mild kyphosis, mild pes valgus and pes planus, no clinodactyly	mild kyphosis, no clinodactyly	no
**Skin features**							
Café-au-lait spots	no	no	no	no	no	no	no
Freckling	no	no	no	no	no	no	no
Other skin/ectodermal features	hyperhidrosis, deep palmar creases	sparse and curly hair, sparse and thin eyebrows and eyelashes, scaly and dry skin, eczematous skin, loose and thick skin, deep palmar creases	nevi	no	n.r.	n.r.	sparse and thin eyebrows and eyelashes, loose skin, deep palmar creases
**Facial features**							
Bitemporal narrowing	yes	yes	yes	yes	yes	yes	yes
Hypertelorism	yes	yes	yes	no	no	no	yes
Low-set and/or posteriorly rotated ears	yes	yes	yes	yes	yes	yes	yes
Prominent nasal bridge	yes	yes	yes	yes	yes	yes	yes
Low posterior hairline	yes	yes	yes	yes	yes	yes	yes
Short/webbed neck	yes	yes	yes	yes	yes	yes	yes
Other dysmorphism or clinical features	high cranial vault, triangular and coarse face, downward slanted palpebral fissures, ptosis, prominent philtrum, large mouth, thick lips, micrognathia, high arched/narrow palate	triangular coarse face, sparse eyebrows, sparse eyelashes, downward slanted palpebral fissures, epicanthus, nasolacrimal duct stenosis, prominent nasolabial sulci, pointed receding chin	helix folding anomaly, dysmorphic ear lobe	downward slanted palpebral fissures, prominent nasolabial folds, long philtrum	downward slanted palpebral fissures, unilateral ptosis	downward slanted palpebral fissures, unilateral ptosis	high forehead, prominent eyes, downward slanted palpebral fissures, thick helix, deeply grooved philtrum, large mouth, thin lips, pointed chin, micrognathia

Abbreviations: F = female; M = male; n.r. = not reported; PVS = pulmonary valve stenosis; ASD = atrial septal defect; Hom = homozygous.

## Data Availability

The data presented in this study are available on request from the corresponding author.

## References

[1] Tartaglia M., Aoki Y., Gelb B.D. (2022). The molecular genetics of RASopathies: An update on novel disease genes and new disorders. Am. J. Med. Genet. C Semin. Med. Genet..

[2] Riller Q., Rieux-Laucat F. (2021). RASopathies: From germline mutations to somatic and multigenic diseases. Biomed. J..

[3] Tajan M., Paccoud R., Branka S., Edouard T., Yart A. (2018). The RASopathy Family: Consequences of Germline Activation of the RAS/MAPK Pathway. Endocr. Rev..

[4] Hilal N., Chen Z., Chen M.H., Choudhury S. (2023). RASopathies and cardiac manifestations. Front. Cardiovasc. Med..

[5] Roberts A.E., Adam M.P., Feldman J., Mirzaa G.M., Pagon R.A., Wallace S.E., Bean L.J.H., Gripp K.W., Amemiya A. (1993). Noonan Syndrome. GeneReviews^®^.

[6] Tartaglia M., Mehler E.L., Goldberg R., Zampino G., Brunner H.G., Kremer H., van der Burgt I., Crosby A.H., Ion A., Jeffery S. (2001). Mutations in *PTPN11*, encoding the protein tyrosine phosphatase SHP-2, cause Noonan syndrome. Nat. Genet..

[7] Motta M., Fasano G., Gredy S., Brinkmann J., Bonnard A.A., Simsek-Kiper P.O., Gulec E.Y., Essaddam L., Utine G.E., Guarnetti Prandi I. (2021). *SPRED2* loss-of-function causes a recessive Noonan syndrome-like phenotype. Am. J. Hum. Genet..

[8] Markholt S., Andreasen L., Bjerre J., Gregersen P.A., Andersen B.N. (2023). Autosomal recessive Noonan-like syndrome caused by homozygosity for a previously unreported variant in *SPRED2*. Eur. J. Med. Genet..

[9] Wakioka T., Sasaki A., Kato R., Shouda T., Matsumoto A., Miyoshi K., Tsuneoka M., Komiya S., Baron R., Yoshimura A. (2001). Spred is a Sprouty-related suppressor of Ras signalling. Nature.

[10] Lorenzo C., McCormick F. (2020). SPRED proteins and their roles in signal transduction, development, and malignancy. Genes Dev..

[11] Lopez J., Bonsor D.A., Sale M.J., Urisman A., Mehalko J.L., Cabanski-Dunning M., Castel P., Simanshu D.K., McCormick F. (2023). The ribosomal S6 kinase 2 (RSK2)-SPRED2 complex regulates the phosphorylation of RSK substrates and MAPK signaling. J. Biol. Chem..

[12] Hirata Y., Brems H., Suzuki M., Kanamori M., Okada M., Morita R., Llano-Rivas I., Ose T., Messiaen L., Legius E. (2016). Interaction between a Domain of the Negative Regulator of the Ras-ERK Pathway, SPRED1 Protein, and the GTPase-activating Protein-related Domain of Neurofibromin Is Implicated in Legius Syndrome and Neurofibromatosis Type 1. J. Biol. Chem..

[13] Yan W., Markegard E., Dharmaiah S., Urisman A., Drew M., Esposito D., Scheffzek K., Nissley D.V., McCormick F., Simanshu D.K. (2020). Structural Insights into the SPRED1-Neurofibromin-KRAS Complex and Disruption of SPRED1-Neurofibromin Interaction by Oncogenic EGFR. Cell Rep..

[14] Kato R., Nonami A., Taketomi T., Wakioka T., Kuroiwa A., Matsuda Y., Yoshimura A. (2003). Molecular cloning of mammalian Spred-3 which suppresses tyrosine kinase-mediated Erk activation. Biochem. Biophys. Res. Commun..

[15] Brems H., Chmara M., Sahbatou M., Denayer E., Taniguchi K., Kato R., Somers R., Messiaen L., De Schepper S., Fryns J.P. (2007). Germline loss-of-function mutations in *SPRED1* cause a neurofibromatosis 1-like phenotype. Nat. Genet..

[16] Legius E., Stevenson D., Adam M.P., Feldman J., Mirzaa G.M., Pagon R.A., Wallace S.E., Bean L.J.H., Gripp K.W., Amemiya A. (1993). Legius Syndrome. GeneReviews^®^.

[17] Arbelo E., Protonotarios A., Gimeno J.R., Arbustini E., Barriales-Villa R., Basso C., Bezzina C.R., Biagini E., Blom N.A., de Boer R.A. (2023). 2023 ESC Guidelines for the management of cardiomyopathies. Eur. Heart J..

[18] McLaren W., Gil L., Hunt S.E., Riat H.S., Ritchie G.R., Thormann A., Flicek P., Cunningham F. (2016). The Ensembl Variant Effect Predictor. Genome Biol..

[19] Richards S., Aziz N., Bale S., Bick D., Das S., Gastier-Foster J., Grody W.W., Hegde M., Lyon E., Spector E. (2015). Standards and guidelines for the interpretation of sequence variants: A joint consensus recommendation of the American College of Medical Genetics and Genomics and the Association for Molecular Pathology. Genet. Med..

[20] Quinodoz M., Peter V.G., Bedoni N., Royer Bertrand B., Cisarova K., Salmaninejad A., Sepahi N., Rodrigues R., Piran M., Mojarrad M. (2021). AutoMap is a high performance homozygosity mapping tool using next-generation sequencing data. Nat. Commun..

[21] den Dunnen J.T., Dalgleish R., Maglott D.R., Hart R.K., Greenblatt M.S., McGowan-Jordan J., Roux A.F., Smith T., Antonarakis S.E., Taschner P.E. (2016). HGVS Recommendations for the Description of Sequence Variants: 2016 Update. Hum. Mutat..

[22] Sund K.L., Zimmerman S.L., Thomas C., Mitchell A.L., Prada C.E., Grote L., Bao L., Martin L.J., Smolarek T.A. (2013). Regions of homozygosity identified by SNP microarray analysis aid in the diagnosis of autosomal recessive disease and incidentally detect parental blood relationships. Genet. Med..

[23] Correia-Costa G.R., Sgardioli I.C., Santos A.P.D., Araujo T.K., Secolin R., Lopes-Cendes I., Gil-da-Silva-Lopes V.L., Vieira T.P. (2022). Increased runs of homozygosity in the autosomal genome of Brazilian individuals with neurodevelopmental delay/intellectual disability and/or multiple congenital anomalies investigated by chromosomal microarray analysis. Genet. Mol. Biol..

[24] Roberts A.E., Allanson J.E., Tartaglia M., Gelb B.D. (2013). Noonan syndrome. Lancet.

[25] Yamamoto G.L., Aguena M., Gos M., Hung C., Pilch J., Fahiminiya S., Abramowicz A., Cristian I., Buscarilli M., Naslavsky M.S. (2015). Rare variants in *SOS2* and *LZTR1* are associated with Noonan syndrome. J. Med. Genet..

[26] Johnston J.J., van der Smagt J.J., Rosenfeld J.A., Pagnamenta A.T., Alswaid A., Baker E.H., Blair E., Borck G., Brinkmann J., Craigen W. (2018). Autosomal recessive Noonan syndrome associated with biallelic *LZTR1* variants. Genet. Med..

[27] Engelhardt C.M., Bundschu K., Messerschmitt M., Renne T., Walter U., Reinhard M., Schuh K. (2004). Expression and subcellular localization of Spred proteins in mouse and human tissues. Histochem. Cell Biol..

